# TIM-3 and CEACAM1 are Prognostic Factors in Head and Neck Squamous Cell Carcinoma

**DOI:** 10.3389/fmolb.2021.619765

**Published:** 2021-07-23

**Authors:** Fan Yang, Ziqing Zeng, Jing Li, Xiubao Ren, Feng Wei

**Affiliations:** ^1^ Department of Immunology, Tianjin Medical University Cancer Institute and Hospital, Tianjin, China; ^2^ Department of Biotherapy, Tianjin Medical University Cancer Institute and Hospital, Tianjin, China; ^3^ National Clinical Research Center for Cancer, Tianjin, China; ^4^ Key Laboratory of Cancer Prevention and Therapy, Tianjin, China; ^5^ Tianjin’s Clinical Research Center for Cancer, Tianjin, China; ^6^ Tianjin Key Laboratory of Cancer Immunology and Biotherapy, Tianjin, China

**Keywords:** HNSCC, TIM-3, CEACAM1, TILs, prognosis

## Abstract

**Background:** T-cell Immunoglobulin and Mucin domain-containing molecule-3 (TIM-3) is a new immune checkpoint molecule which plays important and complex roles in regulating immune responses and in inducing immune tolerance. TIM-3 is expressed on activated T cells and its signaling on cytotoxic T cells leads to T cell exhaustion which is mediated by carcinoembryonic antigen-related cell adhesion molecule 1 (CEACAM1), another well-known molecule expressed on tumor tissues and/or tumor infiltrating lymphocytes (TILs).

**Methods:** In the present study, we investigated TIM-3 and CEACAM1 immunohistochemical expression in 80 head and neck squamous cell carcinoma (HNSCC) specimens, linked to detailed outcome, clinic-pathological parameters. Here we reported scores and absolute counts of TIM-3+/CEACAM1+ TILs, and evaluated the expression of CEACAM1 on tumor tissues.

**Results:** The results showed that more TIM-3+ TILs infiltration correlated with poorer overall survival (*p* < 0.001), as did the presence of CEACAM1 on cancer cells (*p* < 0.001) and CEACAM1+ TILs in tumor microenvironment (*p* = 0.015). Multivariate Cox regression analysis revealed that high TIM-3+ TILs may be considered as an independent prognostic factor of poor disease outcome (hazard ratio, 2.066; 95% confidence interval, 1.027–4.159; *p* = 0.042), as well as cancer cells expressed CEACAM1 level (hazard ratio, 5.885; 95% confidence interval, 2.832–12.230; *p* < 0.001).

**Conclusion:** Our results indicate that expression of TIM-3 and CEACAM1 may represent a highly dysfunctional population of T cells. Our current findings suggest both of them were valuable predicting markers that might provide help for clinicians to design effective immunotherapeutic regimen against head and neck carcinoma.

## Introduction

Head and neck carcinoma (HNC) is a heterogeneous disease including a variety of tumors according to the origin. HNC often occurs in the upper neck, such as the tongue, oropharynx, nasopharynx, and lip. The most common histological subtype of HNC is referred to head and neck squamous cell carcinoma (HNSCC) (Marur and Forastiere, 2016). Despite clinical studies and advance in treatment, satisfactory curative strategy has not yet been reached. Therefore, there is an urgent need for the identification of new potential prognostic markers that predict the clinical outcomes and specific molecular signatures that better serve as suitable therapeutic targets to improve clinical management of patients with HNSCC.

T-cell Immunoglobulin and Mucin domain-containing molecule-3 (TIM-3) is an immune checkpoint molecule discovered in 2002 (Monney et al., 2002). TIM-3 was initially identified as a T cell marker for Th1 and CD8^+^ T cells. It was later shown that TIM-3 is expressed by human Th1 and Th17 cells and also other immune cells encompassing dendritic cells (DCs), macrophages, and natural killer (NK) cells (Ganjalikhani Hakemi et al., 2020). TIM-3 is expressed on activated T cells, and its signaling on cytotoxic T cells leads to reduction in proliferation, decreased production of effector cytokines and apoptosis of effector T cells. TIM-3 mediates its suppressive activity on immune cells via its several ligands including C-type galectin-9 (Gal-9), phosphatidylserine (PtdSer), carcinoembryonic antigen-related cell adhesion molecule 1 (CEACAM1), and high-mobility group protein 1 (HMGB1). Immune checkpoints are critical regulatory pathways of the immune system, and the recent approval of immune-checkpoint blockade strategies has emphasized the importance of regulation of the immune system in cancer development and progression (Tundo et al., 2019). Beyond PD-1/PD-L1 and CD28/CTLA-4 axes, TIM-3 was reported to be an emerging target for cancer immunotherapy. Emerging evidence has shown that deleting TIM-3 in both CD4^+^ and CD8^+^ T cells led to a modest reduction in tumor burden (Dixon et al., 2021). Investigating the expression pattern and the detailed role of TIM-3 in the metabolic alteration of TILs as well as the tumor cells of HNSCC is of critical importance in finding more specific and effective therapeutic approaches in the future.

Carcinoembryonic antigen-related cell adhesion molecules (CEACAMs) are transmembrane glycoprotein belonging to the glycosyphosphatidylinositol-linked immunoglobulin (Ig) superfamily. The human carcinoembryonic antigen (CEA) family has 12 genes belonging to the CEACAM subgroup and has been found to physiologically exist as either soluble forms in body fluids or membrane-bound forms on the apical surface of several cell types, such as endothelial, hematopoietic, and epithelial cells. Depending on the molecular subtype and cell type, CEACAMs regulate cell adhesion, tumor suppression, angiogenesis, activation of immune-reactive cells, and cell cycle. CEACAM1, also known as CD66a or biliary glycoprotein-1, expresses on cell membrane and interacts with its receptors, integrins, and extracellular matrix proteins to modulate the angiogenic and immune responses (Gray-Owen and Blumberg, 2006). These associations are responsible for a multitude of processes that converge into complex and interwoven roles in tumorigenesis and metastatic development. New findings indicated CEACAM1 was an important regulator of CD8^+^ T cell function (Zöller et al., 2020). CEACAM1 was described to be downregulated or overexpressed in numerous tumors, and it has been considered as a promising candidate biomarker for some cancers.

To our knowledge, CEACAM1 has remained unexplored up to the present in HNSCC tissues. Likewise, to what extent TIM-3 is related to HNSCC has not yet been reported. Therefore, to further verify whether CEACAM-1 and TIM-3 were involved in HNSCC progression and their expression profile in HNSCC patients, we measured the expression of CEACAM1 and TIM-3 in HNSCC tissues, and evaluated the combinational clinical significance of CEACAM1 and TIM-3 for the prognosis and treatment decision making in HNSCC. In the present study, CEACAM1 and TIM-3 expression was assessed by immunohistochemistry (IHC) in 80 HNSCCs. We also further examined their correlation with clinical and histopathological features, as well as postoperative survival.

## Results

### Patient Characteristics and Baseline Clinic-Pathologic Characteristics

The study included 80 HNSCC patients who all respectively underwent head and neck carcinoma resection between 2009 and 2013 in TMUCIH and were classified as follows: 17 oropharynx carcinomas, 37 oral cavity/lip carcinomas, 17 larynx carcinomas, and 9 hypopharynx carcinomas. As shown in Table 1, patients were predominantly male (80.0%) and the majority of tumor tissue samples were moderately (58.8%) differentiated. Median age was 56 years (interquartile range, 49–64 years), and most patients had a history of ever alcohol and tobacco use.

**TABLE 1:** Association between TIM-3+/CEACAM1+ TILs infiltration and clinic-pathologic variables in 80 patients with HNSCC.

*Variables*	*Overall*	*TIM-3+ TILs*			*CEACAM1+ TILs*		
		*Low (%)*	*High (%)*	*p value* *^a^*	*Low (%)*	*High (%)*	*p value* *^a^*
***Patient number***	80 (100.0)	41 (51.3)	39 (48.8)		40 (50.0)	40 (50.0)	
***Gender***							
***Male***	64 (80.0)	33 (41.3)	31 (38.8)	0.911	32 (40.0)	32 (40.0)	1.000
***Female***	16 (20.0)	8 (10.0)	8 (10.0)		8 (10.0)	8 (10.0)	
***Age (years)***				0.153			0.491
＜***60***	49 (61.3)	22 (27.5)	27 (33.8)		23 (28.8)	26 (32.5)	
***≥60***	31 (38.8)	19 (23.8)	12 (15.0)		17 (21.3)	14 (17.5)	
***Localization***				0.481			0.785
***Oropharynx***	17 (21.3)	10 (12.5)	7 (8.8)		9 (11.3)	8 (10.0)	
***Non-oropharynx***	63 (78.8)	31 (38.8)	32 (40.0)		31 (38.8)	32 (40.0)	
***Histology***				0.680			0.673
***Well differentiated***	18 (22.5)	8 (10.0)	10 (12.5)		9 (11.3)	9 (11.3)	
***Moderately differentiated***	47 (58.8)	26 (32.5)	21 (26.3)		25 (31.3)	22 (27.5)	
***Poorly differentiated***	15 (18.8)	7 (8.8)	8 (10.0)		6 (7.5)	9 (11.3)	
***T classification***				0.074			0.659
***T1***	23 (28.8)	15 (18.8)	8 (10.0)		14 (17.5)	9 (11.3)	
***T2***	25 (31.3)	15 (18.8)	10 (12.5)		11 (13.8)	14 (17.5)	
***T3***	9 (11.3)	2 (2.5)	7 (8.8)		4 (5.0)	5 (6.3)	
***T4***	23 (28.8)	9 (11.3)	14 (17.5)		11 (13.8)	12 (15.0)	
***Lymph node Metastasis***				0.025			0.818
***Negative (N0)***	49 (61.3)	30 (37.5)	19 (23.8)		24 (30.0)	25 (31.3)	
***Positive (N1-3)***	31 (38.8)	11 (13.8)	20 (25.0)		16 (20.0)	15 (18.8)	
***TNM stage***				0.022			0.923
***Ⅰ***	20 (25.0)	14 (17.5)	6 (7.5)		11 (13.8)	9 (11.3)	
***Ⅱ***	13 (16.3)	9 (11.3)	4 (5.0)		6 (7.5)	7 (8.8)	
***Ⅲ***	11 (13.8)	4 (5.0)	7 (8.8)		6 (7.5)	5 (6.3)	
***Ⅳ***	36 (45.0)	11 (13.8)	22 (27.5)		17 (21.3)	19 (23.8)	
***Tobacco smoking***				0.214			0.348
***Non-smoker***	28 (35.0)	17 (21.3)	11 (13.8)		12 (15.0)	16 (20.0)	
***Smoker***	52 (65.0)	24 (30.0)	28 (35.0)		28 (35.0)	24 (30.0)	
***Alcohol consumption***				0.666			0.823
***Non-drinker***	37 (46.3)	18 (22.5)	19 (23.8)		18 (22.5)	19 (23.8)	
***Drinker***	43 (53.8)	23 (28.8)	20 (25.0)		22 (27.5)	21 (26.3)	
***p16 status***				0.424			0.152
***Negative***	26 (32.5)	15 (18.8)	11 (13.8)		16 (20.0)	10 (12.5)	
***Positive***	54 (67.5)	26 (32.5)	28 (35.0)		24 (30.0)	30 (37.5)	

a *p* values were obtained from the χ^2^ test (two-sided).

### TIM-3+ TILs in Head and Neck Squamous Cell Carcinoma Tissues Were Associated With Patients’ Clinic-Pathological Features and Prognosis

**FIGURE 1:**
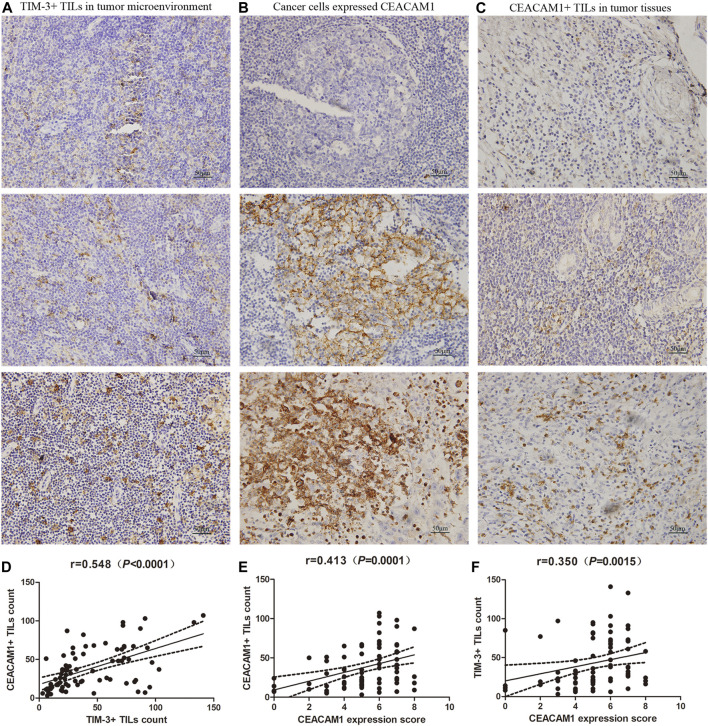
Expressions of T-cell immunoglobulin and domain 3 (TIM-3) and carcinoembryonic antigen-related cell adhesion molecule 1 (CEACAM1) in HNSCC tissues. **(A)** Representative TIM-3 expressions in head and neck squamous cell carcinoma (HNSCC) specimens by immunohistochemistry (×400). **(B)** CEACAM1 was expressed in HNSCC cells, and showed negative staining, positive membrane staining, or strongly cytoplasm staining (×400). **(C)** CEACAM1 was expressed in tumor infiltrating lymphocytes (TILs) (×400). **(D)** The number of TIM-3+ TILs was positively related to that of CEACAM1 (*r* = 0.548, *p*＜0.001). **(E)** CEACAM1+ TILs count was positively associated with the CEACAM1 expression level in cancer tissues (*r* = 0.413, *p* ＜ 0.001). **(F)** TIM-3+ TILs count demonstrated a significantly positive correlation with the expression of CEACAM1 in cancer cells (*r* = 0.350, *p* = 0.001).

We conducted an evaluation of TIM-3 staining (Figure 1A) and correlation with clinic-pathological parameters on 80 HNSCC patients. We observed a majority of HNSCC cases (91.3%) with intratumoral and/or stromal TIM-3+ TILs (≥10). The relationship between TIM-3+ immune cells infiltration and the clinic-pathological features of HNSCC was shown in Table 1. According to the Chi-square test, TIM-3 expression was observed to be closely related to tumor size, lymph node metastasis, and TNM stage. However, it was not related to gender, age, tumor location, tumor differentiation degree, history of tobacco smoking or alcohol consumption, and p16 status.

The median follow-up duration was 39.5 months (ranging from 2 to 103 months). At the end of the follow-up period, there were 47 deaths and 33 survivals. The overall median survival time of the 80 HNSCC patients was 47 months. Among the 39 patients with high TIM-3+ TILs infiltration, 24 months was revealed as the median survival time. The 41 patients with low TIM-3+ TILs infiltration were observed to have a median survival time of 66 months. The 1-, 3-, 5-years OS rate for all enrolled patients with high TIM-3+ TILs infiltration was 79.5, 33.1, and 21.1%, respectively, and 95.1, 72.6, and 70.1% for patients with low TIM-3+ TILs infiltration. The difference of survival time between them was statistically significant (Log-rank rest, χ^2^ = 20.021, *p* < 0.001). The 1, 3, and 5-years DFS rate for patients with high TIM-3+ TILs infiltration was 51.3, 28.2, and 22.2%, respectively, and 85.4, 65.3, and 60.3% for patients with low TIM-3+ TILs infiltration (Log-rank rest, χ^2^ = 16.636, *p* < 0.001). These all indicated that high TIM-3+ TILs infiltration led to significantly worse prognosis postoperatively.

Since TIM-3+ TILs infiltration was associated with TNM stage, we further performed survival analysis in patients with separate clinical stage. For stage Ⅲ and Ⅳ, patients with high TIM-3+ TILs infiltration had shorter OS and DFS time than those with low TIM-3+ TILs infiltration (Log-rank rest, all *p* < 0.001). For stage Ⅰ and Ⅱ, however, the difference of survival time between high TIM-3+ TILs infiltration group and low TIM-3+ TILs infiltration group was not significant (*P*_OS_ = 0.991, *P*_DFS_ = 0.899).

### CEACAM1 Expression Level in Cancer Samples Was Correlated With the Number of TIM-3+ TILs in Patients With Head and Neck Squamous Cell Carcinoma

Based on the results of TIM-3 staining, we continued to evaluate the expression of CEACAM1 on both HNSCC cells and tumor infiltrating lymphocytes to get a more comprehensive understanding of their roles in HNSCC progression (Figures 1B,C). Positive CEACAM1 expression was mainly observed in the membrane and cytoplasm of carcinoma cells. Figure 1B showed representative immunohistochemical staining of CEACAM1 in the membrane and/or cytoplasm of HNSCC cells. As the results shown by Table 2, compared to well-differentiated squamous cell carcinoma with more membranous expression, the intermediately and poorly differentiated squamous cell carcinoma demonstrated more cytoplasmic expression (*p* = 0.005). Also, intermediately and poorly differentiated squamous cell carcinoma demonstrated stronger CEACAM1 expression than well-differentiated carcinoma (*p* = 0.041). On the other hand, the relationship between CEACAM1+ immune cells infiltration and the clinic-pathological features of HNSCC was shown in Table 1. We found that the number of CEACAM1+ immune cells infiltration was not influenced by clinic-pathological variables such as gender, age, tumor location, tumor differentiation degree, tumor size, lymphatic invasion, TNM stage, smoking or drinking status, and p16 status (all *p* > 0.05).

**TABLE 2:** Number of cancer tissues analyzed for CEACAM1 expression in HNSCC.

*Parameter*	*Patients (n)*	*CEACAM1 Median *(*P25, P75*)	*Expression patterns*		
			*Membranous*	*Cytoplasmic*	*Negative*
***Patient number***	80 (100.0)	5.00 (4.00, 6.00)	24	52	4
***Gender***					
***Male***	64 (80.0)	6.00 (4.25, 7.00)	16	45	3
***Female***	16 (20.0)	5.00 (3.00, 5.75)	8	7	1
***Age (years)***					
＜***60***	49 (61.3)	5.00 (4.00, 7.00)	16	32	1
***≥60***	31 (38.8)	6.00 (3.00, 6.00)	8	20	3
***Localization***					
***Oropharynx***	17 (21.3)	5.00 (4.00, 6.50)	8	8	1
***Oral cavity/Lip***	37 (78.8)	6.00 (4.00, 6.50)	11	25	1
***Larynx***	17 (21.3)	5.00 (4.00, 6.50)	4	11	2
***Hypopharynx***	9 (11.3)	6.00 (4.00, 6.50)	1	8	0
***Histology***					
***Well differentiated***	18 (22.5)	4.50 (2.75, 6.00)	11	5	2
***Moderately differentiated***	47 (58.8)	5.00 (5.00, 6.00)	10	35	2
***Poorly differentiated***	15 (18.8)	6.00 (4.00, 7.00)	3	12	0
***T Classification***					
***T1***	23 (28.8)	5.00 (4.00, 6.00)	9	12	2
***T2***	25 (31.3)	5.00 (3.50, 6.50)	7	16	2
***T3***	9 (11.3)	6.00 (5.00, 7.00)	2	7	0
***T4***	23 (28.8)	6.00 (5.00, 7.00)	6	17	0
***Lymph node metastasis***					
***Negative (N0)***	49 (61.3)	5.00 (3.00, 6.00)	20	25	4
***Positive (N1-3)***	31 (38.8)	6.00 (5.00, 7.00)	4	27	0
***TNM stage***					
***Ⅰ***	20 (25.0)	5.00 (4.00, 6.00)	8	10	2
***Ⅱ***	13 (16.3)	4.00 (2.50, 6.00)	5	6	2
***Ⅲ***	11 (13.8)	6.00 (5.00, 6.00)	4	7	0
***Ⅳ***	36 (45.0)	6.00 (5.00, 7.00)	7	29	0
***Tobacco smoking***					
***Non-smoker***	28 (35.0)	5.00 (3.25, 6.00)	10	17	1
***Smoker***	52 (65.0)	5.50 (4.25, 6.75)	14	35	3
***Alcohol consumption***					
***Non-drinker***	37 (46.3)	5.00 (3.50, 7.00)	12	23	2
***Drinker***	43 (53.8)	6.00 (4.00, 6.00)	12	29	2
***p16 status***					
***Negative***	26 (32.5)	5.00 (4.00, 6.00)	9	15	2
***Positive***	54 (67.5)	6.00 (4.00, 7.00)	15	37	2

To investigate the relationship of cancer cells expressed CEACAM1 level and the number of CEACAM1+ TILs infiltration, correlation analysis was performed at all stage patients. The results showed that the CEACAM1+ TILs count was positively associated with the CEACAM1 expression level in cancer tissues (Figure 1D, *r* = 0.413, *p*<0.001). Next, we explored whether TIM-3 and CEACAM1 expressions were correlated. We found that the number of TIM-3+ TILs was positively related to that of CEACAM1 (Figure 1E, *r* = 0.548, *p*<0.001). Moreover, we analyzed the correlation between TIM-3+ TILs infiltration and cancer tissue expressed CEACAM1. As shown in Figure 1F, TIM-3+ TILs count demonstrated a significantly positive correlation with the expression of CEACAM1 in cancer cells (Figure 1F, *r* = 0.350, *p* = 0.001).

### Prognostic Significance of CEACAM1 Expression in Head and Neck Squamous Cell Carcinoma Tissues

**FIGURE 2:**
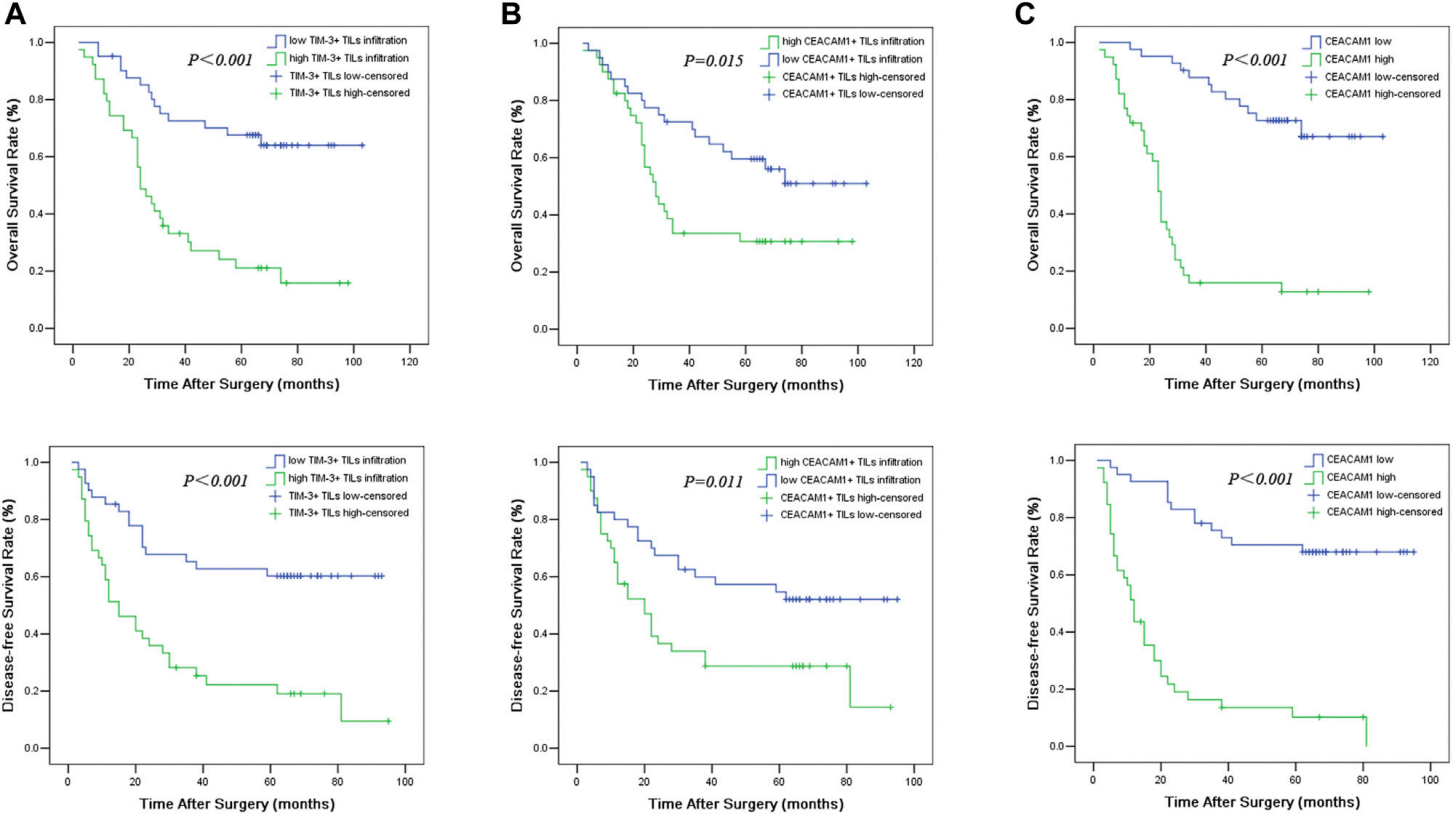
Overall survival (OS) and disease-free survival (DFS) curves of HNSCC patients according to TIM-3+ TILs infiltration **(A)**, CEACAM1+ TILs infiltration **(B)**, and cancer expressed CEACAM1 levels **(C)**.

The overall Kaplan-Meier survival curves for CEACAM1 expression on both HNSCC cells and tumor infiltrating lymphocytes in cancer tissues were shown in Figure 2. In univariate analyses, the presence of CEACAM1+ TILs in tumor tissues was linked to poor overall survival and disease-free survival (*P*_OS_ = 0.015, *P*_DFS_ = 0.011). Similarly, high expression for CEACAM1 in cancer tissue was suggested to have an unfavorable influence on OS and DFS (*P*_OS_ < 0.001, *P*_DFS_ < 0.001).

Univariate analysis showed that OS has significant correlation with TNM stage (*p* = 0.001), tumor size (*p* = 0.019), lymph node metastasis (*p* < 0.001), TIM-3+ TILs infiltration (*p* < 0.001), CEACAM1+ TILs count (*p* = 0.015), and cancer cells expressed CEACAM1 (*p* < 0.001), while DFS was directly influenced by histologic grading (*p* = 0.045), TNM stage (*p* = 0.001), tumor size (*p* = 0.016), lymph node metastasis (*p* = 0.002), TIM-3+ TILs infiltration (*p* < 0.001), CEACAM1+ TILs count (*p* = 0.011), and cancer cells expressed CEACAM1 (*p* < 0.001). Multivariate analysis demonstrated that lymph node metastasis, TIM-3+ TILs infiltration, and CEACAM1 expression were independent prognostic factors for OS, while lymph node metastasis and CEACAM1 expression could be regarded as independent prognostic factors of DFS (all *p* < 0.05, Tables 3 and 4). Patients with low CEACAM1 expression on tumor cells and low CEACAM1+ TILs infiltration had a more favorable outcome.

**TABLE 3:** Univariate and multivariate analysis of clinicopathologic factors in HNSCC patients with respect to OS.

*Characteristics*	*Cases,* *n* (%)	*Univariate*		*Multivariate*	
		*Survival time (month)*	*p* *value*	*HR (95% CI)*	*p* *value*
***Gender***					
***Male***	64 (80.0)	56.23 ± 5.04	0.419		
***Female***	16 (20.0)	61.94 ± 8.54			
***Age (years)***					
＜***60***	49 (61.3)	54.45 ± 5.33	0.594		
***≥60***	31 (38.8)	61.46 ± 7.33			
***Localization***					
***Oropharynx***	17 (21.3)	46.30 ± 7.24	0.998		
***Non-oropharynx***	63 (78.8)	58.50 ± 5.00			
***Histology***					
***Well differentiated***	18 (22.5)	72.75 ± 8.51	0.058		0.278
***Moderately differentiated***	47 (58.8)	54.83 ± 5.51			
***Poorly differentiated***	15 (18.8)	37.20 ± 6.57			
***TNM stage***					
***Ⅰ/Ⅱ***	33 (41.3)	74.07 ± 6.07	0.001		0.183
***Ⅲ/Ⅳ***	47 (58.8)	45.54 ± 5.38			
***Tumor size***					
***≤4 cm***	48 (60.0)	64.28 ± 5.41	0.019		0.687
＞***4 cm***	32 (40.0)	46.03 ± 6.59			
***Lymph node metastasis***					
***Negative (N0)***	49 (61.3)	71.03 ± 5.64	0.000	2.111 (1.111–4.013)	0.023
***Positive (N1-3)***	31 (38.8)	37.65 ± 5.35			
***Tobacco smoking***					
***Non-smoker***	28 (35.0)	57.30 ± 7.02	0.676		
***Smoker***	52 (65.0)	56.87 ± 5.53			
***Alcohol consumption***					
***Non-drinker***	37 (46.3)	54.55 ± 6.10	0.827		
***Drinker***	43 (53.8)	58.81 ± 6.04			
***p16 status***					
***Negative***	26 (32.5)	65.90 ± 7.52	0.120		
***Positive***	54 (67.5)	53.33 ± 5.30			
***TIM-3+ TILs count***					
***Low***	41 (51.3)	76.98 ± 5.76	0.000	2.066 (1.027–4.159)	0.042
***High***	39 (48.8)	37.85 ± 5.08			
***CEACAM1+ TILs count***					
***Low***	40 (50.0)	69.39 ± 6.10	0.015		0.152
***High***	40 (50.0)	45.81 ± 5.76			
***CEACAM1***					
***Low***	41 (51.3)	83.67 ± 4.80	0.000	5.885 (2.832–12.230)	0.000
***High***	39 (48.8)	30.80 ± 4.59			

**TABLE 4:** Univariate and multivariate analysis of clinicopathologic factors in HNSCC patients with respect to DFS.

*Characteristics*	*Cases*, *n* (%)	*Univariate*		*Multivariate*	
		*Survival time (month)*	*p* *value*	*HR (95% CI)*	*p* *value*
***Gender***					
***Male***	64 (80.0)	45.10 ± 4.94	0.284		
***Female***	16 (20.0)	57.19 ± 9.34			
***Age (years)***					
＜***60***	49 (61.3)	47.35 ± 5.67	0.891		
***≥60***	31 (38.8)	42.65 ± 5.81			
***Localization***					
***Oropharynx***	17 (21.3)	42.37 ± 8.12	0.805		
***Non-oropharynx***	63 (78.8)	47.20 ± 4.92			
***Histology***					
***Well differentiated***	18 (22.5)	70.94 ± 9.19	0.045		0.234
***Moderately differentiated***	47 (58.8)	41.35 ± 5.14			
***Poorly differentiated***	15 (18.8)	32.20 ± 7.43			
***TNM stage***					
***Ⅰ/Ⅱ***	33 (41.3)	63.90 ± 6.40	0.001		0.206
***Ⅲ/Ⅳ***	47 (58.8)	35.92 ± 5.29			
***Tumor size***					
***≤4 cm***	48 (60.0)	55.91 ± 5.73	0.016		0.572
＞***4 cm***	32 (40.0)	34.91 ± 6.20			
***Lymph node metastasis***					
***Negative (N0)***	49 (61.3)	56.92 ± 5.56	0.002	2.253 (1.237–4.102)	0.008
***Positive (N1-3)***	31 (38.8)	31.48 ± 5.99			
***Tobacco smoking***					
***Non-smoker***	28 (35.0)	51.14 ± 7.55	0.640		
***Smoker***	52 (65.0)	44.36 ± 5.18			
***Alcohol consumption***					
***Non-drinker***	37 (46.3)	48.32 ± 6.56	0.964		
***Drinker***	43 (53.8)	45.48 ± 5.70			
***p16 status***					
***Negative***	26 (32.5)	57.75 ± 7.99	0.114		
***Positive***	54 (67.5)	42.48 ± 5.07			
***TIM-3+ TILs count***					
***Low***	41 (51.3)	63.77 ± 5.85	0.000		0.094
***High***	39 (48.8)	29.76 ± 5.10			
***CEACAM1+ TILs count***					
***Low***	40 (50.0)	59.61 ± 6.17	0.011		0.753
***High***	40 (50.0)	35.09 ± 5.57			
***CEACAM1***					
***Low***	41 (51.3)	73.21 ± 5.15	0.000	7.143 (3.623–14.085)	0.000
***High***	39 (48.8)	20.43 ± 3.89			

## Discussion

Unfortunately, HNSCC is an aggressive tumor with high incidence of metastasis and recurrence rate, and the 5-years survival rate is also low, especially for the advanced cases. Growing evidences have indicated that tumor can induce a persistent chronic immune response in most cases which may trigger the T cell exhaustion (Pauken and Wherry, 2015), and subsequently failure to eliminate antigen. T cell exhaustion is usually associated with co-expression of high levels of multiple inhibitory receptors, such as PD-1 and others. The PD-1 blockage has been implicated in clinical trials and shown encouraging results. Currently, FDA approved antibodies against PD-1 for second-line therapy of recurrent and/or metastatic HNSCC. However, the benefits and improved prognosis for HNSCC patients remain unsatisfactory (Ho and Mehra, 2019). These findings indicate that there are other promising targets that are involved in T cell exhaustion and impairment of immune response. In our present work, we have likely to investigate the other two potential targets that may be applied in immunotherapy for the treatment of HNSCC, named TIM-3 and CEACAM1.

Recently, accumulating studies have indicated TIM-3 to be a putative antitumor negative mediating factor due to its preferential expression on the exterior of activated Th1 cells. T cell inhibitory receptor TIM-3 is expressed during exhausted T cell differentiation. Although there have been a number of studies investigating the expression of TIM-3 in various tumors (Peng et al., 2017; Zhang et al., 2017; Burugu et al., 2018), the results remain inconclusive in HNSCC, and the relationship between TIM-3 expression and prognosis remains unclear. In our study, TIM-3+ TILs infiltration was detected in HNSCC tissues by immunochemistry. We observed TIM-3+ TILs (≥10) was present in about 91.3% of cases. Moreover, the presence of TIM-3+ TILs was associated with tumor size, lymph node metastasis, and TNM stage. In prognostic analyses, HNSCC patients with low TIM-3+ TILs infiltration had significantly improved survival for all assessed endpoints, as compared to patients with high TIM-3+ TILs. In multivariate analyses, the prognostic effect of TIM-3+ TILs infiltration was maintained. Our study has been consistent with other reported studies in finding that a high TIM-3 expression is closely related to tumor proliferation, invasion, metastasis and clinical prognosis. Similar results have been confirmed in gastric (Shen et al., 2016), liver (Liu et al., 2018), pancreatic (Peng et al., 2017), colorectal (Zhang et al., 2017). However, another study in breast cancer has shown the exact opposite. Burugu et al. (Burugu et al., 2018) investigated TIM-3 immunohistochemical expression in 3,992 early breast cancer specimens. They finally found the presence of TIM-3+ intra-epithelial TILs as an independent favorable prognostic factor. Indeed, we found that in early-stage HNSCC, the predicting role and the relationship between the number of TIM-3+ TILs with patients’ survival was not obvious. All the above may suggest that TIM-3 expressed in TILs contributes to the aggressiveness of the late-stage tumor, as it does in gastrointestinal carcinomas.

To date, co-expression of TIM-3 and CEACAM1 in HNSCC has not yet been investigated. Therefore, in our present work the potential role of them as possible biomarker of HNSCC were investigated. Figures 1D,F showed that TIM-3+ TILs highly correlated with co-infiltration of CEACAM1+TILs and CEACAM1 expression on carcinoma cells. Results were consistent with other reported studies and implied that TIM-3 was co-expressed with CEACAM1 and involved in T-cell inhibition. What’s more, Huang et al. (2015) found that CEACAM1-deficient T cells were hyperinflammatory with reduced cell surface expression of TIM-3 and regulatory cytokines, and this was restored by T-cell-specific CEACAM1 expression. This suggests that CEACAM1 serves as a heterophilic ligand for TIM-3 expressed on activated T cells that is required for its ability to mediate T-cell inhibition. In other words, TIM-3 and CEACAM1 form an axis that can inhibit immune responses, thereby downregulating their antitumor immunity.

Unlike TIM-3, CEACAM1 plays a more complex role in tumorigenesis. CEACAM1 is considered as an adhesion molecule that governs the growth and differentiation of normal or cancerous tissue. Depending on tumor type and histologic grading, CEACAM1 have apparently opposite actions, including tumor-suppressive or tumor-promoting function. Detailed investigations of its expression patterns in different tumors are crucial for diagnosis, prognosis, and treatment. CEACAM1 was reported to be a growth inhibitor in a number of early solid neoplasms, such as liver cancer, bladder cancer, and colon cancer, etc (Zhang et al., 2017). In addition, CEACAM1 was down-regulated in 30% of breast cancers compared to more than 90% of colon cancers, suggesting CEACAM1 delivered a strong growth inhibitory signal upon its expression in fully differentiated epithelial cells (Li et al., 2016). In this case, CEACAM1 expression seems to function as a tumor suppressor. Many epithelial malignancies could overcome this inhibition by down-regulating the CEACAM1 gene. However, our findings apparently contradict such a function because of CEACAM1 overexpression in HNSCC. This has also been questioned by other findings: CEACAM1 is overexpressed in some malignancies including non-small cell lung cancer (Dango et al., 2008), melanoma (Ortenberg et al., 2014), gastric carcinoma (Zhou et al., 2009), and metastatic colon cancer (Ieda et al., 2011). Moreover, accumulating studies suggest that CEACAM1 is gradually along tumor development and progression. Recent studies have shown that CEACAM1 regulates CD8^+^ T cell immunity and induces T cell exhaustion (Zöller et al., 2020). Consistent with the above results, our data showed that the expression of CEACAM1 was higher in more advanced stages, especially in stage Ⅲ/Ⅳ respect to stage Ⅱ, indicating its promotional role in HNSCC metastasis. Meanwhile, according to our results, CEACAM1 expression was higher in poorly differentiated carcinomas compared with well differentiated ones, which implies its correlation with unfavorable prognosis. Further analysis showed CEACAM1 expression on carcinoma cells was an independent prognostic factors for both OS and DFS. What need to be noted is that there was a CEACAM1 expression shift from membrane to cytoplasm from well differentiated to poorly differentiated squamous cell carcinoma. Our results indicated that membranous expression pattern of CEACAM1 might function as a tumor suppressor in well differentiated squamous cell carcinoma. In our opinion, cytoplasmic CEACAM1 might not function as a tumor suppressor and be related to tumor invasion and progression. Similar to our results, the function of CEACAM1 has been confirmed in ovarian tumors (Li et al., 2016), oral tumors (Wang et al., 2017) and laryngeal squamous cell carcinoma (LSCC) (Lucarini et al., 2019).

We hold the opinion that the present work has several strong characteristics. We have examined the expression of two potential targets (TIM-3 and CEACAM1) which might be applied to immunotherapy of HNSCC. There were also some limitations in our study. Firstly, we investigated the expression of TIM-3 and CEACAM1 only in patients having opportunity to receive surgical therapy. For those HNSCC patients with distant metastasis, the data was unavailable and needs to be further studied. Secondly, the expression of TIM-3 and CEACAM1 in normal head and neck (oral/oropharyngeal/nasopharyngeal/labial/lingual) epithelium and primary benign epithelial tumors were not determined in our study. Therefore, substitutive tests in normal epithelium and benign epithelial tumors are required to confirm the importance of TIM-3 and CEACAM1 in tumorigenesis of HNSCC. Furthermore, new findings indicated the expression of TIM-3 and its ligand CEACAM1 on both CD4^+^ and CD8^+^ TILs could protect T cells from apoptosis, promote the expansion of the immunosuppressive cell population, but also induce T cell exhaustion (Zöller et al., 2020; Dixon et al., 2021; Yang et al., 2021), suggesting that anti-TIM-3/CEACAM1 axis therapy is promising candidate for combination with other therapeutic modalities diminish regulatory T cells or co-stimulate CD8 T cells to treat head and neck carcinoma. We need further work to explore the specific mechanism.

Taken together, our results indicate that expression of TIM-3 and CEACAM1 may represent a highly dysfunctional population of T cells, and we prove that their high expressions in tumor tissues predict poor prognosis of patients with HNSCC, especially for advanced ones. Furthermore, the presence of TIM-3+ TILs is positively correlated with the number of CEACAM1+ TILs in tumor microenvironment and CEACAM1 expression in HNSCC tissues. All the current findings suggest that both TIM-3 and CEACAM1 were valuable predicting markers that might provide help for clinicians to design effective immunotherapeutic regimen against head and neck carcinoma.

## Materials and Methods

### Sample Collection

This study was approved by the Human Ethic Committee of Tianjin Medical University *Cancer* Institute and Hospital (TMUCIH), Tianjin, China. In addition, the procedures were in accordance with the Helsinki Declaration of 1975. Written informed consent was obtained from each patient before enrollment. Eighty formalin-fixed paraffin-embedded HNSCC samples were collected from the Institute of Pathological Anatomy of TMUCIH, Tianjin, China, between August 2009 and October 2013. Following surgical removal, all the tissue samples were fixed in formalin and embedded in paraffin prior to sectioning for histological analyses. The TNM staging system was used to classify the tumors in accordance with the American Joint Committee on *Cancer* classification. All the diagnoses were made by at least two pathologists. All patients were followed up until December 2018, in other words, all patients were followed for at least 5 years after surgery, and any locoregional recurrences, distant metastasis, and death events were recorded. The clinical features of these patients were summarized in Table 1.

### Immunohistochemistry

Primary antibodies used for IHC of HNSCC tissues included Human TIM-3 Affinity Purified Polyclonal Antibody (1:100, AF 2365, R&D Systems, Minneapolis, United States), and CEACAM1 (D3R8O) Rabbit Monoclonal Antibody (1:200, Cat# 44,464, Cell Signaling Technology, United States). Anti-goat secondary antibody labeled by HRP and DAB immunohistochemistry kit (PV-9003) was purchased from Beijing Zhongshan Jinqiao Biological Technology Co., Ltd.

Briefly, slides were dewaxed, and endogenous peroxidase was blocked by immersing the slides in a 3% solution of hydrogen peroxide in methanol for 20 min. This was followed by a step of antigen retrieval; the slides were immersed in 0.01 M/L citrate buffer solution (pH 9.0) and placed in a microwave oven for 25 min. Following a wash in 1-M phosphate-buffered saline (PBS, pH 7.4), the sections were covered with normal serum in a humidity chamber for 30 min at room temperature. Excess serum was rinsed off with 1 M PBS, and the sections were incubated with the primary antibodies at 4 °C overnight in a humidity chamber. After that the sections were rinsed with PBS before incubation with the biotinylated second antibody for 30 min at 37°C in a humidity chamber. After rinsing with PBS, the streptavidin–peroxidase complex reagent (StrepABComplex/HRP Duet, DAKO) was added. Slides were incubated for 30 min at RT, washed in 1 mol PBS, and covered with 3,3′-diaminobenzidine tetrahydrochloride solution for 15 min under microscope. Sections were then immersed in running tap water, counterstained with hematoxylin for 1 min, followed by tap water bath, immersion in alcohol steps, then xylene, and then applied the coverslips. The primary antibody was omitted in the negative controls.

### Evaluation of Immunohistochemistry Staining

Histologic and IHC evaluation were performed by three pathologists independently. For CEACAM1, the method of Kawai et al. (Wang et al., 2017) was used to calculate a semiquantitative score. The intensity score of staining was scored into four categories according to the color of immune reaction: 0, negative; 1, light brown; 2, brown; and 3, dark brown. The proportion of positively stained tumor cells was evaluated and scored as: 0, none; 1, Ì1/100; 2, 1/100–1/10; 3, 1/10–1/3; 4, 1/3–2/3; 5, Î2/3 tumor cells. The intensity and proportion score were then added together resulting in a total score of 0–8 for each staining. Results were expressed as the median (Percentiles 25, Percentiles 75) for each group. A final score of 0–1 was considered as negative expression (-, noted as 0); 2–3: weak positive expression (+, noted as 1); 4–5: moderate positive expression (++, noted as 2); and 6-8 was strong positive expression (+++, noted as 3). The median score of CEACAM1 was 5. In view of this, negative, weak or moderate positive expression was defined as low expression, and strong positive expression was defined as high expression for CEACAM1.

TIM-3+/CEACAM1+ lymphocytes scores were reported as absolute counts per core in the tumor microenvironment. The positive cells per core were counted manually and average counts of five fields were used for analysis.

### Statistical Analysis

All statistical analyses were performed using Statistical Package for the Social Sciences version 17.0 (IBM Corp., United States), and the normality of the data was evaluated using the Kolmogorov-Smirnov test. The median numbers of TIM-3 and CEACAM1+ TILs were used as a cutoff point to define the TILs-low and TILs-high groups. Chi-square (χ^2^) test were used to compare categorical variables. The correlations between CEACAM1 expression and TIM-3+ TILs infiltration were assessed using Spearman’s correlation analysis. Kaplan-Meier analysis was the method used to calculate the overall survival (OS) and disease-free survival (DFS), then the OS and DFS was compared by log-rank test. Multivariable analysis was performed by the Cox proportional hazard model. Statistically significant was considered as two-tailed *p* < 0.05.

## Data Availability

The original contributions presented in the study are included in the article/Supplementary Material, further inquiries can be directed to the corresponding author.
